# Disparities in access to systemic therapies for patients with hepatocellular carcinoma: an analysis from the International Liver Cancer Association

**DOI:** 10.1016/j.lanepe.2025.101408

**Published:** 2025-07-31

**Authors:** Manon Allaire, Leonardo G. Da Fonseca, Marco Sanduzzi-Zamparelli, Won-Mook Choi, Cecilia Monge, Ken Liu, Michael Leibfried, Sarah Manes, Zorana Maravic, Milan Mishkovikj, Jordi Bruix, Helen L. Reeves, Riad Salem, Bruno Sangro

**Affiliations:** a AP-HP Sorbonne Université, Hôpital Universitaire Pitié-Salpêtrière, Service d’Hépato-gastroentérologie, Paris, France; b INSERM UMR 1138, Centre de recherche des Cordeliers, Paris, 75006, France; c Clinical Oncology, Instituto do Cancer do Estado de Sao Paulo, University of São Paulo School of Medicine, Brazil; d BCLC Group, Institut d'Investigacions Biomèdiques August Pi i Sunyer (IDIBAPS), Barcelona, Spain; e Centro de Investigación Biomédica en Red en Enfermedades Hepáticas y Digestivas (CIBEREHD), Madrid, Spain; f Department of Gastroenterology, Liver Center, Asan Medical Center, University of Ulsan College of Medicine, Seoul, Republic of Korea; g Thoracic and Gastrointestinal Malignancies Branch, National Cancer Institute, National Institutes of Health, MD, USA; h AW Morrow Gastroenterology and Liver Centre, Royal Prince Alfred Hospital, Sydney, New South Wales, Australia; i Faculty of Medicine and Health, Sydney University, Sydney, New South Wales, Australia; j Genentech, South San Francisco, CA, USA; k Global Liver Institute Washington District of Columbia, USA; l Digestive Cancers Europe, Brussels, Belgium; m European Liver Patient Association (ELPA), Brussels, Belgium; n Newcastle University Translational Research Institute and Newcastle upon Tyne NHS Foundation Trust, United Kingdom; o Department of Radiology, Section of Interventional Radiology, Northwestern University, Chicago, IL, USA; p Liver Unit and HPB Oncology Area, Clinica Universidad de Navarra and CIBEREHD, Pamplona–Madrid, Spain; q Liver Oncology Unit, Liver Unit, Hospital Clínic, Barcelona, Spain

**Keywords:** Hepatocellular carcinoma, Systemic therapies, Disparities, Healthcare, Atezolizumab-bevacizumab, Durvalumab-tremelimumab, Sorafenib, Lenvatinib, Inequality, Inequity, Liver cancer, Reimbursement

## Abstract

Hepatocellular carcinoma (HCC) is a major cause of cancer deaths worldwide. It is often diagnosed at advanced stages, requiring systemic therapies like sorafenib, lenvatinib, atezolizumab-bevacizumab, and tremelimumab-durvalumab. However, disparities in access to systemic therapies for HCC remain a major global challenge, particularly in low- and middle-income countries where high drug costs, regulatory delays, and limited healthcare infrastructure impede treatment. Drawing on international experience, we highlight the urgent need for coordinated advocacy and innovative programs—such as Project Orbis and expedited approval pathways—to improve drug availability. Multisectoral collaboration among patient groups, clinicians, policymakers, and industry is essential to ensure equitable access to life-saving therapies and to reduce the burden of HCC worldwide.

**Search strategy and selection criteria**

References for this analysis were identified through searches of PubMed and official websites of regulatory and health agencies using the terms (“hepatocellular carcinoma” OR “HCC”) AND (“systemic therapies” OR “cancer drug access” OR “reimbursement” OR “regulatory approval”) from January 2005 through March 2025. Additional sources were identified through the review of key national and international cancer treatment guidelines, publications by organizations such as the World Health Organization, and direct data contributions from pharmaceutical companies. Only articles and documents published in English were reviewed. The final selection of references was based on their relevance to the global disparities in access to systemic therapies for hepatocellular carcinoma and the scope of this Personal View.

## Introduction

Hepatocellular carcinoma (HCC), a major cause of cancer-related deaths globally, is projected to reach 1.4 million cases annually by 2040, with the largest increases expected in low- and medium-development countries.^1^ Strategies to reduce HCC's impact should prioritize prevention, early diagnosis, and access to effective treatments. The International Liver Cancer Association (ILCA) has emphasized challenges in early detection, resource equity, and the critical role of health authorities in managing HCC.^2^^,^^3^

Despite well-known risk factors and screening recommendations, around 50% of patients are still diagnosed with HCC at advanced stages and many of them reach these stages after progression to local therapies. Here, systemic treatment may extend survival and delay clinical deterioration.^4^ Four systemic regimens have been approved by at least one regulatory agency for the treatment of HCC (Table 1). The tyrosine kinase inhibitors sorafenib and lenvatinib were first approved by the Food and Drug Administration (FDA) for HCC in November 2007 and August 2018, respectively. The immunotherapy combinations of atezolizumab plus bevacizumab and tremelimumab plus durvalumab were approved by the FDA in May 2020 and October 2022, respectively.^5^, ^6^, ^7^, ^8^ Very recently, ipilimumab plus nivolumab has also shown survival benefit in advanced stage HCC.^9^ Furthermore, signals of efficacy have also been demonstrated in earlier stages in combination with transarterial chemoembolization (for durvalumab plus bevacizumab and lenvatinib plus pembrolizumab), although mature survival data are still not available.^10^^,^^11^ Clinical trials for advanced HCC are limited to select populations with preserved liver function, restricted geographic representation, and offer survival gains measured in months. High development costs also lead to expensive therapies. HCC patients face significant challenges, including high mortality, poor quality of life, and coexisting liver diseases like cirrhosis. Access to treatment remains a major issue globally, with the burden being particularly severe in low- and medium-resource countries.^2^ In many countries with a marked incidence of HCC, health systems fragmented between public and private sectors often result in incomplete coverage and deficient health infrastructures, as in some Latin American nations.^12^^,^^13^ Furthermore, inconsistent drug pricing, complex regulatory processes, delays in the approval of novel drugs with proven efficacy and reimbursement policies are observed.^14^, ^15^, ^16^, ^17^, ^18^, ^19^, ^20^, ^21^ The situation is particularly critical for patients living in low- and middle-resource countries, where access to many of the medicines listed on the World Health Organization (WHO) Essential Medicines List may be lacking.^22^^,^^23^ In the absence of government reimbursement and insurance coverage, many individuals have to pay out-of-pocket health expenses, leading to unequal access, treatment interruption, and financial toxicity. To date, no study has specifically focused on highlighting the current disparities in access to systemic therapy for liver cancer.

**Table 1:** Characteristics of the studied therapies.

	Sorafenib	Lenvatinib	Atezolizumab-bevacizumab	Durvalumab-tremelimumab
Treatment administration plan	Twice a day oral, continuous administration	Once a day oral, continuous administration	Every 3 weeks IV infusion	Every 4 weeks IV infusion
Drug class	Inhibits multiple tyrosine kinases	Inhibits multiple tyrosine kinases	Atezolizumab inhibits PD-L1 and Bevacizumab inhibits VEGF	Tremelimumab inhibits CTLA-4 and Durvalumab inhibits PD-L1
Mechanism of action	Antiproliferative and antiangiogenic	Antiproliferative and antiangiogenic	Immunestimulating and antiangiogenic	Immunestimulating
Primary endpoint of the pivotal phase III trial that supports prescription	Superior survival compared to placebo	Non-inferior survival compared to sorafenib	Superior survival compared to Sorafenib	Superior survival compared to Sorafenib
Date of FDA approval	November 2007	August 2018	May 2020	October 2022
Indication as per the FDA label	Unresectable hepatocellular carcinoma	For the first-line treatment of patients with unresectable hepatocellular carcinoma	Unresectable or metastatic hepatocellular carcinoma	Unresectable hepatocellular carcinoma

Aligning the medical community, government, pharmaceutical industry, and civil society is key to fostering equitable HCC care. Advocacy is essential in providing support, raising community awareness, and enhancing care quality by creating a more supportive environment for patients.^24^, ^25^, ^26^ This approach aids early detection and helps patients navigate healthcare systems. Advocacy relies on collaboration to advance HCC care and influence policies. This article explores systemic treatment accessibility and regional disparities. Specifically, we focus on differences in the process for approval and reimbursement decisions while the cost-effectiveness issue exceeds the aim of this manuscript. The high costs of cancer treatment are well documented, with final pricing determined through negotiations between pharmaceutical companies and national regulatory agencies, with each country defining how they will govern commerce. The development of new agents carries a relevant investment that the industry expects to recover, with a mean cost of development of $ 4.4 billion per drug.^27^ This corporate goal may collide with the patients' ability to pay or reimburse agency budgets. The development of generics affects the realm of patent ownership, but unfortunately, this strategy has not improved global treatment access in a significant way. Specific access programs in defined areas may provide some relief, but a major impact may come from reducing industry costs, with innovative trial design, the use of artificial intelligence, and the incorporation of virtual twin controls.^28^ Additionally, a positive impact may come from promoting value-based pricing, ensuring adequate analysis of validated endpoints in clinical trials for approval purposes, and investing in translational research to optime personalized strategies. These promising approaches are still developing and are expected to gain prominence in the future. Similarly, achievements in protein structure prediction and theoretical chemistry—recognized with a Nobel Prize—require further refinement before full integration into clinical cancer research and pricing models. Given the complexity of cost and cost-effectiveness issues, that also affects other non-cancer diseases, our objectives here aimed at displaying the heterogeneity in approval and reimbursement processes, advocating equitable patient access across populations.

## Disparities in healthcare systems across regions

**Fig. 1:**
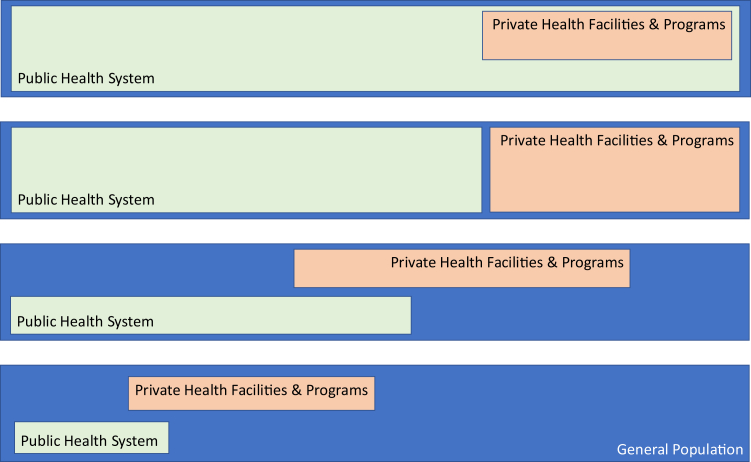
**Integration of the public and private sectors in different models of healthcare**.

Healthcare in most countries combines public and private systems (Fig. 1). Public systems, tax-funded and government-managed, aim for universal care but face challenges like underfunding, limited infrastructure, and resource disparities, particularly in rural areas. Private systems, funded by individuals and insurers, often provide faster services with better facilities but limited coverage. Medication availability and affordability depend on system performance and regulatory processes. For analysis, we examine three countries per continent, plus Australia, categorized by income levels based on World Bank classifications.^29^ North, Central, and South America are treated as one continent in this analysis. The countries analyzed were selected because they are representative of their specific continents. Information was readily available and trustable from recognized physicians/investigators that are members of ILCA. If the information was not available through ILCA membership, the needed details where obtained from the pharmaceutical companies as mentioned in the paragraph “Access to systemic therapies for HCC according to the type of treatment and region”. A more granular analysis including all countries was not considered. Table 2 summarizes the healthcare systems in selected countries, with data on system design and drug reimbursement factors sourced from literature and official healthcare websites.

**Table 2:** Characteristics of the healthcare systems in the selected countries from different continents.

Continent	Countries	Liver cancer age standardized incidence per 100,000	Health care system	World Bank classification by income	UHC coverage	Health index score 2023	Current health expenditure in US dollars per capita	Current health expenditure (% of GDP)
America	United States	6.8	Multi-payer system with no universal coverage	High	86	73.3	11.702.41	17%
	Brazil	4.5	Hybrid public universal coverage and private multi-payer system	Upper-middle	80	71.7	700.71	10%
	Bolivia	6.7	Multi-payer system with no universal coverage	Low-middle	65	66	241.12	7%
Europe	France	7.8	Universal coverage with single-payer system	High	85	80.5	6.629.56	12.2%
	Spain	6.0	Universal coverage with single-payer system	High	85	79.7	2.900.65	9%
	Romania	8.9	Universal coverage with multi-payer system	High	78	73	809.59	6%
Asia	Japan	9.2	Universal coverage with multi-payer system	High	83	86.5	5.2510.63	10.9%
	Republic of Korea	13.7	Universal coverage with multi-payer system	High	89	84.8	3.031.18	8%
	India	2.7	Multi-payer system with no universal coverage	Low-middle	63	66.2	56.63	3%
Africa	South Africa	4.9	Hybrid public and private multi-payer system with no universal coverage	Upper-middle	71	59.9	489.64	9%
	Egypt	32.0	Hybrid private and public multi-payer system with no universal coverage	Low-middle	70	67.2	150.91	5%
	Angola	6.6	Multi-payer system with no universal coverage	Low-middle	37	49.9	51.00	2.9%
Australia	Australia	7.2	Universal coverage with multi-payer system	High	87	80.4	5.901.11	10%

Liver Cancer incidence according to Globocan 2022 (version 1.1) 08.02.2024. http://gco.iarc.who.int/today.

UHC stands for Universal Health Coverage. It is a global health policy goal that aims to ensure that all people have access to essential health services without suffering financial hardship. UHC service coverage index combines 14 tracer indicators of service coverage into a single summary measure (https://data.who.int/indicators/).

Legatum health index is on a scale of 0 (lowest) to 100 (highest)∗. The Legatum Prosperity Index is a system that rates nations on how well they support the flourishing of their citizens. Taking into account both economic and social well-being. It goes beyond conventional macroeconomic measures of a country's prosperity. Which rely primarily on indications of wealth like average income per person (GDP per capita). It reflects the richness of a truly prosperous existence (https://www.statista.com/statistics/1290168/health-index-of-countries-worldwide-by-health-index-score/).

Health expenditure per capita. Year 2022 in US dollars: World Health Expenditure database (apps.who.nha.database) retrieved on April 7, 2023.

https://data.worldbank.org/indicator/SH.XPD.CHEX.PC.CD.

### Africa: South Africa, Egypt, and Angola

Africa has an age-standardized HCC incidence rate per 100,000 of 8.4, with Egypt (32.2) and the Gambia (23.9) having the highest rates in the region.^30^ The etiology varies across regions. For HCC, it is mostly related to hepatitis B virus (HBV) infection (45%) in Angola, while alcohol consumption is the most common cause (40%) in South Africa, and hepatitis C virus (HCV) infection is the leading cause (44%) in Egypt.^3^ HBV vaccination is not systematically implemented in African countries, and aflatoxin B1, which originates from fungal contaminations of staple foodstuffs, is a relevant co-factor for HCC development in Central and Southern Africa.^31^^,^^32^

In South Africa, with an upper-middle-income economy, the healthcare landscape reflects a complex interplay of historical, socioeconomic, and political factors.^29^ Public and private healthcare systems operate together, with 80% of the population relying on the underfunded, tax-supported public system, while 80% of physicians work in the better-equipped private sector. The National Health Insurance (NHI) aims to improve equity by redistributing resources, integrating sectors, upgrading public facilities, negotiating better drug prices, and monitoring healthcare quality.

In Egypt, a low-middle-income country, healthcare is provided through a mix of public and private systems. The government-run Egyptian HealthCare Authority offers basic services to most of the population, but underfunding, limited infrastructure, and unequal resource distribution create disparities, especially in rural areas. Private healthcare provides access to a wider range of amenities. The Universal Health Insurance (UHI), funded by government, employer, and employee contributions, aims to integrate public and private sectors, upgrade facilities, train professionals, and negotiate better drug pricing.

Angola, another low-middle-income country, has significant internal disparities. Better-equipped healthcare is concentrated in cities like Luanda, while advanced cancer treatments are largely unavailable. Private facilities offer higher-quality services. The National Health Development Plan (PNDS 2012–2025), funded by government and international organizations, seeks to improve infrastructure, funding, and workforce training, but faces challenges such as resource shortages and infectious disease burdens. Collaborative programs aim to harmonize cancer guidelines and enhance care standards.^33^^,^^34^

### Asia: South Korea, Japan, and India

In Asia, HCC represents a significant health burden, accounting for 72.5% of global cases in 2020,^35^ with HBV and HCV infections being the primary risk factors. While HBV vaccination and HCV prevention efforts have led to a slight decline in HCC incidence rates, there is a notable rise in cases attributed to metabolic factors such as metabolic syndrome, obesity, and metabolic dysfunction-associated steatotic liver disease (MASLD), signaling a shifting epidemiological trend in recent years.^36^ Asian healthcare systems differ greatly in structure, funding, and accessibility, significantly affecting cancer medication access and affordability.

South Korea, classified as a high-income country, presents one of the most advanced healthcare systems in Asia, thanks to its successful implementation of a comprehensive social health insurance framework that extends coverage to approximately 97% of the population.^29^ In South Korea, the National Health Insurance Service (NHIS) provides universal coverage for all residents, including eligible foreign nationals, funded through income-based premiums collected via monthly taxes. The NHIS covers 50%–80% of essential medical expenses, including accidents, diseases, and maternity care, while cancer patients pay only 5% out-of-pocket, with the rest covered by the NHIS. Government subsidies are available for those facing financial hardship. Many South Koreans also opt for private supplementary insurance for broader coverage and to manage out-of-pocket costs. This sector is highly regulated, with premiums determined by factors like age and gender, and limited employer flexibility in plan design.

Japan, a high-income country, provides universal health insurance ensuring access to essential healthcare for all citizens and residents. The system mandates enrollment for everyone and includes schemes tailored to employees, the self-employed, and the elderly. Insurance premiums are income-based, shared between employers and employees, with support for low-income individuals. Patients can choose providers, as most accept public insurance. Co-payments are generally 30%, with caps to limit out-of-pocket expenses.

In India, the healthcare system operates through a mixed model of public and private health insurance, regulated by the Insurance Regulatory and Development Authority. India is a low-middle-income country with a public healthcare expenditure of 2.1% of GDP in 2021–22.^29^ Government-run facilities provide free or subsidized care, including secondary, tertiary, and primary health centers, while private hospitals in urban areas offer advanced care. Health costs are covered by government schemes, private insurance, or out-of-pocket payments, which remain significant for many. Health insurance coverage rose to 35% in 2018, covering 514 million people by 2021. The 2018 Ayushman Bharat initiative improves access and financial protection, offering free medicines, teleconsultations, and services through Health and Wellness Centers and the Pradhan Mantri Jan Arogya Yojana.^37^ The Central Government, through the National Program for Prevention and Control of Non-Communicable Diseases (NCD), has established 753 District NCD Clinics, State Cancer Institutes, and 20 Tertiary Care Cancer Centres. These facilities, integrated into the National Health Mission, provide cancer and NCD diagnosis and treatment across various healthcare levels, including District Hospitals, Medical Colleges, AIIMS, Central Government Hospitals, and private institutions.

### Australia

In Australia, HCC is the 12th most commonly diagnosed cancer and the 6th most common cause of cancer-related death.^38^ Its incidence has increased almost four-fold over the last 40 years and it is the fastest growing cause of cancer mortality.^39^ Viral infections remain the major cause of HCC in Australia, with more than 50% of HCC patients born overseas. Indigenous Australians, constituting about 3% of the population, present poor prognosis when diagnosed with HCC, with 2.4 times higher rates of incidence and mortality compared to non-Indigenous populations.^39^ This disparity arises from various factors, including limited access to healthcare services resulting in delayed presentation, higher prevalence of risk factors for liver disease, socio-economic disadvantages, and the geographical remoteness of Indigenous communities across Australia.^40^ Australia is a high-income country. The public sector offers universal health coverage through Medicare, which provides subsidized or free services (hospital and medical services, test imaging, and scans) to residents regardless of where they live or their ability to pay.^29^^,^^41^ Despite comprehensive coverage, rural and remote communities face healthcare access disparities. The Pharmaceutical Benefits Scheme (PBS) subsidizes prescription medication costs, but access to specialized or high-cost drugs remains challenging due to regulatory hurdles. HCC patients, however, receive 100% coverage through Medicare.

### Europe: France, Spain, and Romania

Liver cancer incidence and mortality rates vary across Europe, with an incidence that accounts for 10% of HCC worldwide.^32^ Higher HCC rates have been reported in economically disadvantaged areas. Alcohol is one of the most significant risk factors for liver cancer in many Eastern European countries, the United Kingdom and France. In recent years, there has also been an increase in MASLD-related HCC.^42^ Despite HBV vaccination and HCV treatment efforts, HBV and HCV infections remain frequent causes of HCC. This is partly due to the arrival of migrants and refugees from countries with high to intermediate endemicity for these infections.^42^ An EU citizen can use healthcare services in any EU country (without pre-authorization in emergencies, with pre-authorization through insurance in other cases).

In France, a high-income country, social health insurance stands as a pillar of its universal healthcare coverage, funded primarily through taxes and social security contributions.^43^ France's healthcare system provides free access to services like consultations, hospital stays, and medications, with substantial government subsidies. Out-of-pocket payments in France are the lowest in the EU (<8.9% vs. 15.4% average), as public and private insurance cover most costs.^44^ Patients with HCC will be fully covered at 100% for the care related to their condition. Currently, there are increasing disparities between urban and rural areas, with a shortage of medical staff in the countryside compared to urban areas, resulting in significant delays for access to specialized consultations in rural areas.

Spain, a high-income country, has a decentralized public healthcare system managed by 17 autonomous regions, leading to regional variations in accessibility. Funded through taxes, it ensures universal care for citizens and legal residents, allowing cancer patients choice of physicians and specialists. About 30% of the population holds private insurance, contributing to 20% of healthcare spending. While government subsidies make medications affordable, regional disparities in healthcare professionals, wait times, and cancer outcomes persist, emphasizing the need for improved cancer care.

Romania, a high-income country, navigates a mixed healthcare system, blending public and private provisions to deliver basic medical services to all citizens.^29^ Health spending per capita is the lowest among EU countries with a cancer expenditure accounted for 7.1% of total health spending (EUR 160 per capita, compared with an EU average of EUR 326^45^). Nearly 80% of healthcare spending comes from the public system, funded by mandatory insurance contributions. However, it faces underfunding, poor infrastructure, and staff shortages, especially in rural areas. Cancer care is free under the National Oncology Program, but quality lags behind the EU average, with barriers like limited screening, delayed diagnoses, slow access to new therapies, and minimal outpatient services.

### Latin America: Bolivia and Brazil

In Latin America, a high proportion of HCC patients are diagnosed at advanced stages, with an increasing impact of MASLD in HCC occurrence, but still a major role for viral infections.^46^^,^^47^ Moreover, alcohol should be recognized as a potential risk factor for HCC, particularly in MASLD patients who may underreport their alcohol consumption.^48^ Healthcare systems exhibit a notable degree of heterogeneity, reflecting the diverse socioeconomic, political, and cultural landscapes of the region. Disparities in healthcare infrastructure, funding, and service delivery contribute to differential access to medical resources and treatments.^49^ Variations in drug access create challenges in ensuring equitable availability and affordability, with regulatory frameworks influencing approval speed and efficiency.

In Brazil, an upper-middle-income country, universal health coverage is provided by the Unified Health System (SUS), funded by local, state, and federal governments. Complementing SUS, private institutions serve about 25% of the population through private insurance and out-of-pocket payments.^50^ Generally, novel therapies and technologies are more effectively and timely adopted into the private sector, while their availability in the public sectors is hampered by high incorporation costs.

Bolivia is a low-middle income country and faces numerous healthcare challenges, reflecting socioeconomic factors and historical developments. The country has a mixed public-private healthcare system, with the public sector divided into two subsystems: the contributory social security system and the non-contributory public system, provided by the Ministry of Health to the population that cannot access the contributory subsystem.^51^ Private health care is accessible to a very small percentage of the Bolivian population.

### United States of America

The incidence and mortality of HCC in the United States increased from 1970 to 2010, followed by a mortality plateau and a slight decrease per year.^52^ This scenario is likely to be related to the changing risk factors distribution, with a decrease in viral hepatitis. On the other hand, MASLD is an increasingly common cause of HCC and the fastest growing cause of HCC in liver transplant candidates.^52^^,^^53^ Disparities in access to cancer treatment in the United States are multifaceted and influenced by various factors such as race, ethnicity, and socioeconomic status.^54^ Studies have shown that health disparities in chronic liver disease, disproportionately affect socially disadvantaged populations.^55^ Factors like tumor presentation and treatment-related variables have been identified as contributors to racial and ethnic survival disparities in HCC patients.^56^ Additionally, the impact of geographic disparities on access to cancer clinical trials highlights the complexity of ensuring equitable access to care across different regions despite the US being classified as a high-income country.^57^

## Drug approval and reimbursement processes

**Fig. 2:**
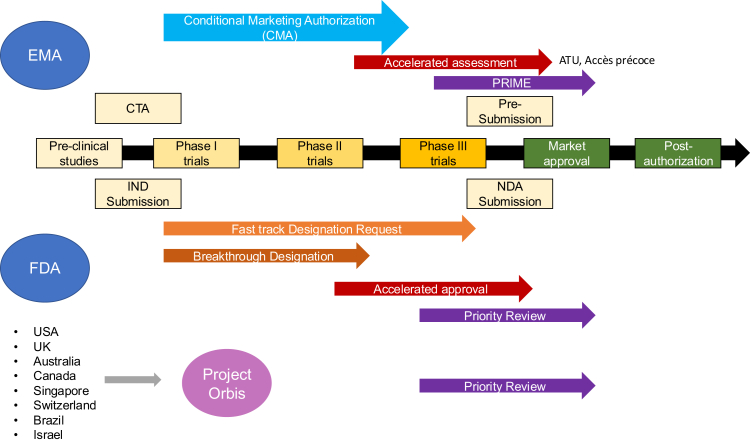
**General process of drug approval and strategies to improve access to effective therapies**. The figure shows the general steps involved in drug approval according to FDA and EMA. In the figure, some of the strategies (ATU, Project Orbis or NASWSI) currently used in or across countries to faster the approval are also shown. ATU: Autorisation Temporaire d'Utilisation; CMA: conditional marketing authorization; CTA: clinical trial application; EMA: European Medicines Agency; FDA: Food and drug association; IND: investigational new drug; NDA: New drug application.

Patient access to therapies relies on regulatory approval, governed by bodies like the International Council for Harmonization (ICH), established in 1990 to harmonize global standards. The drug approval process includes five steps: discovery and development (testing compounds for efficacy, dosage, and safety), preclinical research (animal testing for safety and efficacy), clinical research (human trials across four phases), a thorough application review to ensure safety and effectiveness, and post-authorization surveillance to monitor real-world use. These processes are largely standardized among major regulatory agencies (Fig. 2).

Significant disparities persist despite efforts to harmonize drug approval processes across regions. Beyond approval, patient access often depends on favorable evaluations by health technology assessment (HTA) agencies, which are crucial for drug reimbursement by public health systems and insurers. HTA capacity and reimbursement decision-making vary widely across and within countries. These assessments frequently face delays of months to years after regulatory approval. A summary of key regulatory agency processes is provided.

### Food and Drug Administration

The U.S. FDA oversees drug and device marketing authorizations, typically approving new agents within 6–10 months. It collaborates with applicants to refine labeling and monitors post-market safety through adverse event reporting and facility inspections to ensure manufacturing compliance. The FDA regulates drug advertisements to include accurate prescribing information and must approve changes to applications, such as labeling or dosage. In cases of safety concerns or lack of confirmatory data, the FDA may restrict use or revoke conditional approval.

Reimbursement for drugs involves a mixed system of public and private coverage and is not dependent on HTA assessments after approval. Private insurance is often provided by employers, with employee contributions, and covers approximately two thirds of patients. Private plans may vary as to the prescription medications that are covered and they share costs with the patients. Government health programs provide medications to specific groups, such as the elderly and disabled (Medicare) and low-income individuals (Medicaid). Mechanisms to negotiate drug prices play a crucial role, such as pharmacy benefit managers (PBM), affecting the reimbursement amount. Despite the existence of public and private insurance, approximately 28 million people lack coverage in the United States.^58^

### European Medicines Agency

The European Medicines Agency (EMA) oversees medication approvals within the European Economic Area, including EU member states, Iceland, Liechtenstein, and Norway. It evaluates and authorizes medicines via a centralized process, allowing companies to submit a single marketing application for approval across member states. The Committee for Medicinal Products for Human Use (CHMP) assesses applications and recommends marketing approval. Final authorization is granted by the European Commission within 67 days. However, reimbursement decisions are made at the national level, leading to significant variability in access. Germany is often prioritized by pharmaceutical companies for product launches due to its large market and automatic reimbursement following EMA approval.^59^ Countries requiring cost-effectiveness evaluations by HTAs, such as the UK and the Netherlands, or lengthy negotiations with national and regional authorities, like Spain and Italy, face delays in drug access and limited uptake within two years of launch. If domestic patients are excluded from multi-regional clinical trials, additional local trials may be needed, further delaying approval, particularly in underrepresented countries.^60^, ^61^, ^62^, ^63^ Delays in drug access can have serious consequences. After EMA approval of ipilimumab for melanoma and abiraterone for prostate cancer, 67,000 patients missed treatment in the first year, leading to an estimated 21,600 life-years lost. The longer EMA process compared to the FDA resulted in an additional loss of 8693 life-years.^59^

The EU Health Technology Assessment (HTA) Regulation (2022) aims to harmonize the evaluation of medicines across EU member states. By integrating regulatory and reimbursement processes at the central level, it seeks to streamline HTA, expedite therapy market entry, and reduce assessment duplication. A joint clinical assessment framework ensures uniform, transparent evaluations, fosters collaboration among national HTA agencies, and leverages regulatory approval evidence for reimbursement decisions.

### Pharmaceuticals and Medical Devices Agency

The Pharmaceuticals and Medical Devices Agency (PMDA) in Japan collaborates with the Ministry of Health, Labor and Welfare to review marketing authorization applications, monitor post-market safety, and compensate for adverse drug reactions. It evaluates products against scientific and ethical standards, conducting regulatory consultations and inspections. PMDA reviewers, with expertise in fields such as medicine, biostatistics, and pharmaceutical science, assess drug products, supported by external expert consultations. Review reports are submitted to the Ministry, which has final approval authority.

### Therapeutic Goods Administration

In Australia, the Therapeutic Goods Administration (TGA) regulates drugs through pre-market assessments, post-market monitoring, and licensing manufacturers. Applications are reviewed by scientists and clinicians for quality, safety, and efficacy using a risk-based approach. Lower-risk medicines undergo certification checks, while higher-risk medicines require detailed evaluation, taking 8–11 months based on priority. Approved drugs are added to the Australian Register of Therapeutic Goods for supply and prescription. Subsidization is not automatic; the Pharmaceutical Benefits Advisory Committee, an independent body, reviews medicines three times annually for inclusion in the PBS based on clinical effectiveness, safety, and cost-effectiveness.

### Drug approval processes in Latin America

Medication approval in Latin America varies by country but generally follows a similar framework. Regulatory agencies like ANVISA (Brazil), ISP (Chile), and INVIMA (Colombia) grant market authorizations and oversee post-market safety. Some countries also assess efficacy, safety, cost-effectiveness, and budget impact to determine reimbursement. Brazil includes public consultation with stakeholders in this process. Timelines and requirements differ, but some nations streamline approvals by recognizing EMA or FDA authorizations.

## Access to systemic therapies for HCC according to the type of treatment and region

**Fig. 3:**
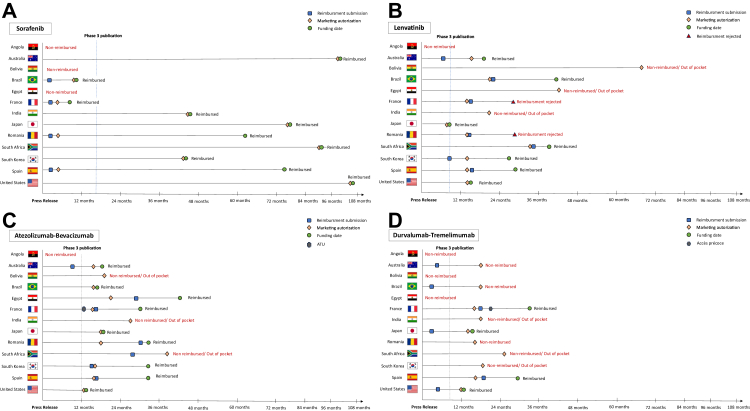
**Timeline of the approval process for (A) Sorafenib, (B) Lenvatinib, (C) Atezolizumab-Bevacizumab, and (D) Durvalumab-Tremelimumabthe four therapies with an approved indication for the treatment of HCC**.

To dissect the reimbursement process for advanced HCC drugs shown effective in phase III trials, data from companies like Roche, AstraZeneca, Bayer, and Eisai, along with public sources, were analyzed. Disparities in regulatory timelines are detailed in Table 3 and Fig. 3. Sorafenib, the first treatment to show a survival benefit in the SHARP trial, demonstrated a median survival of 10.7 months compared to 7.9 months with placebo.^8^ Sorafenib, approved for HCC in November 2007, faced a 13-month gap between trial results and publication, and over 36 months for reimbursement in 8 of 13 studied countries. Only France and Brazil achieved reimbursement within 12 months, while Angola, Bolivia, and Egypt, all lower-middle-income countries, still lack reimbursement, and patients have to pay to have access to this treatment. After failure of several other drugs in demonstrating superiority, lenvatinib proved non-inferiority vs. sorafenib in the REFLECT trial with a median survival of 13.6 months for lenvatinib and 12.3 months for sorafenib.^5^ The FDA approved lenvatinib for HCC in August 2018, 19 months after the press release and 7 months before full results publication. Of 13 countries analyzed, reimbursement was achieved in only 7, while France and Romania rejected it. Atezolizumab plus bevacizumab was the first combination to show superior survival over sorafenib in the IMbrave 150 trial.^6^ The FDA approved the combination in May 2020, eight months after the press release and one month after full results publication. Reimbursement followed within 36 months in most countries but remains unavailable in Angola and unreimbursed in Bolivia, India, and South Africa. Tremelimumab plus durvalumab also was shown to be superior over sorafenib in terms of overall survival in the HIMALAYA trial.^7^ It was approved by the US FDA in October 2022, 12 months after the press release and 4 months after the publication of the full results. Currently, tremelimumab plus durvalumab is reimbursed in 17 countries worldwide.

**Table 3:** Time to submission, approval, and reimbursement of the four therapies with an approved indication for the treatment of HCC.

	Low-middle income countries				Upper-middle income countries		High income countries						
	Angola	Bolivia	Egypt	India	Brazil	South Africa	Australia	France	Japan	Romania	South Korea	Spain	United States
Sorafenib													
Submission	na	na	na	na	1.8	na	na	2.9	na	3.1	na	2.9	na
Approval	na	na	na	46.5	9.7	88.8	103	4.2	79.9	4.2	41.7	4.2	106.4
Reimbursement	Not reimbursed	Not reimbursed	Not reimbursed	46.5	9.6	88.8	103	8.4	79.9	62.3	41.7	74.3	106.4
Lenvatinib													
Submission	na	na	na	na	21.7	35.4	8.9	14.8	na	13.8	9.9	15.7	na
Approval	na	68.4	44.3	19.7	29.7	34.4	14.8	13.8	8.9	13.8	13.8	13.8	13.8
Reimbursement	Not reimbursed	Not reimbursed	Not reimbursed	Not reimbursed	41.3	40.3	20.7	Not reimbursed	8.9	27.6	27.6	29.5	13.8
Atezo-beva													
Submission	na	na	29.9	na	na	27.9	9.3	17.1	na	30.9	17.1	19.1	na
Approval	na	19.7	21.3	38.7	14.5	39.7	15.0	17.1	16.9	17.1	14.1	17.1	12.1
Reimbursement	Not reimbursed	Not reimbursed	45.6	Not reimbursed	14.5	Not reimbursed	16.1	26.9	16.9	34.7	34.7	34.7	12.1
Durva treme													
Submission	na	na	na	na	7,39	na	6.4	18.9	4.3	na	na	na	6.1
Approval	na	na	na	19.2	19.2	24.7	19.8	15.9	13.3	15.9	19.2	15.9	11.9
Reimbursement	Not reimbursed	Not reimbursed	Not reimbursed	Not reimbursed	Not reimbursed	Not reimbursed	Not reimbursed	32.9	16.7	Not reimbursed	Not reimbursed	30.0	11.9

Time is expressed in months. **na**: not available.

Submission: time from press release to marketing authorization approval (MAA) submission.

Approval: time from press release to market authorization.

Reimbursement: time from press release to reimbursement by healthcare authorities.

Access to therapies is often delayed by slow reimbursement in many countries, unlike the swift access in the US and Japan. Notably, FDA review times showed no correlation with the clinical benefit of new cancer drugs.^64^ This underscores the importance of additional efforts to strengthen the criteria for expedited programs, aiming to facilitate the swift approval of new cancer drugs demonstrating high clinical benefit and acceptable safety risks.^65^, ^66^, ^67^ Of note, in Bolivia, none of the current standards of care first-line therapies (sorafenib, lenvatinib, atezolizumab-bevacizumab, and tremelimumab-durvalumab) are registered in the National List of Essential Drugs.^68^ This is relevant since regions with limited access to HCC treatments often experience poorer outcomes,^69^ underscoring the need to raise awareness about the heterogeneity in approval and reimbursement processes. This highlights the importance of streamlining these processes to prevent inequities caused by unnecessary delays.

## Strategies to improve access to effective therapies

Since 2012, regulatory agencies worldwide have increasingly implemented expedited programs alongside existing frameworks to accelerate the approval of new cancer drugs.^14^^,^^64^ However, their performance varies greatly from one to another. In an analysis of expedited cancer drug approvals by the FDA in the US, EMA in the EU, PMDA in Japan, SwissMedic in Switzerland, Health Canada in Canada, or TGA in Australia from January 2007 to May 2020, 80% of new cancer drugs were first approved by the FDA, while only 8% received their first approval from the EMA and 9% by the PMDA.^64^ Priority review rates for new cancer drugs were 82% at the FDA, 17% at the EMA, and 14% at the PMDA, with median review times of 6.1, 9.7, and 10.5 months, respectively. For FDA-approved drugs, subsequent approvals took a median of 9.7 months at the EMA and 37.4 months at the PMDA, largely due to delays in regulatory submissions.

These findings highlight the complex interplay of application submissions, regulatory frameworks, expedited review processes, and reimbursement policies, as well as access to health care system, resulting in significant and varied barriers to HCC therapy access. All stakeholders share responsibility and must collaborate to address these challenges.

Initiatives like the EU HTA Regulation aim to speed up cancer drug approvals through joint submissions and rapid reviews. Similarly, the New Chemical Entities Work Sharing Initiative, launched in 2018 by Australia, Canada, Switzerland, and Singapore, collaborates on medication approvals, reducing regulatory burden and aligning authorization timelines. Early approvals include apalutamide for prostate cancer, abemaciclib for breast cancer, and niraparib for ovarian and related cancers.

*Project Orbis* was launched in 2019 as a parallel review program and is coordinated by the US FDA alongside regulatory agencies from the UK, Australia, Canada, Singapore, Switzerland, Brazil, and Israel. Any partner may propose products for inclusion in the scheme, and although the FDA usually reaches a regulatory decision first, each partner remains fully independent on their final regulatory decision. As an example, the submission gap and review time for oncology applications at Swissmedic were significantly reduced by participation in *Project Orbis*.^70^ The performance of the program was recently analyzed by Swissmedic by comparing outcomes of marketing authorization applications for cancer drugs submitted through or outside of *Project Orbis* in 2020 and 2021. *Project Orbis* resulted in shorter submission gap (time between submission at the FDA and Swissmedic, median 33 vs. 168 days) and review time at Swissmedic (235 vs. 314 days), and similar rates of Swissmedic approval (77% vs. 76%) and consensus decisions (81% vs. 76%).

Bridging approval and reimbursement is vital for early therapy access. In France, programs like ATU and Accès Précoce enabled early use of HCC immunotherapies before full authorization or reimbursement. These initiatives, guided by strict safety evaluations, allowed specific patients access to treatments like atezolizumab-bevacizumab and durvalumab-tremelimumab under close monitoring.

However, all that glitters is not gold and the benefits of earlier access should be balanced against futile use when expedite review processes are considered. Accelerated approvals (AA) in oncology are frequently based on surrogate endpoints such as progression-free survival or response rate. Although the correlation among such surrogates and overall survival is poor, less than 50% of the confirmatory trials granted regular approval after the demonstration of overall survival improvement.^71^ Specifically, in a study of cancer drugs granted AA by the FDA in the last decade, for a total of 129 cancer drug–indication pairs, only 43% demonstrated a clinical benefit in confirmatory trials while 63% were converted to regular approval, 22% were withdrawn, and 15% remained ongoing after a median of 6.3 years.^72^ Interestingly, among medications converted to regular approval, less than 70% of them showed improvements in overall survival or quality of life. Priority should be given to fast-tracking agents with survival benefits proven in phase III trials over those based on weaker evidence, such as phase II trials or non-validated surrogate endpoints like PFS or RFS. Indeed, a surrogate endpoint should be valid regardless of the intervention and must reliably predict survival.^73^, ^74^, ^75^ Considering that the majority of patients with liver cancer also have underlying liver disease, the risk of liver decompensation and its associated mortality must also be taken into account. This is why the most recent EASL guidelines, issued in 2024, recommend using overall survival as the sole valid endpoint for clinical trials involving systemic therapies.^76^ Therefore, physicians and patient advocacy groups should primarily focus on ensuring access to treatments with clear survival advantages.

In addition, a long history of legal disputes between India's generic drug industry and pharmaceutical companies raises a critical question: does patent protection contribute to systemic HCC treatment inequities, particularly in low-income countries where affordability and accessibility heavily rely on generic alternatives?^77^ Patent protection and drug accessibility often conflict, and diluting patents to improve access may not be a viable solution, given the substantial drawbacks associated with weakening patent protections. Notably, Gilead's non-exclusive, royalty-free licensing agreements with local manufacturers for generic HIV and HBV drugs in developing countries offer a potential model to follow in liver cancer, although some may argue that it is a strategy to maintain market monopoly. While controversial, whether this model could be adapted to systemic HCC treatment warrants further discussion.

Lastly, while improving and expediting access to systemic therapies is a potential strategy to improve HCC outcomes, continued efforts should also be made at the other end of the disease spectrum, *i.e.,* HCC prevention and its early detection. Currently there are no countries with a structured program to screen for liver fibrosis at a national level. However, several projects evaluating this approach at a population level are underway in the United States (Renown), and Europe (Scarred Liver Project, LiverScreen, and SEAL).^78^ When implemented, this screening has the potential to identify patients with chronic liver disease at an early stage to be treated with disease modifying therapies (such as antiviral drugs, alcohol cessation programs, and recent drugs for fibrotic MASLD) and in turn prevent hepatocarcinogenesis.^79^ With regards to early detection of HCC, the aforementioned countries and regions all have local guidelines with recommendations on HCC surveillance.^4^^,^^76^^,^^80^, ^81^, ^82^, ^83^, ^84^, ^85^ Despite this, surveillance utilization remains suboptimal overall.^86^ Indeed, some Asian countries (*e.g.,* Japan, South Korea, and Taiwan) have adopted national HCC surveillance programs which has led to earlier diagnosis and improved survival compared to Western countries without such programs.^87^ Clearly, the absence of national programs for early detection of liver fibrosis and HCC, even in high-income countries, represents a significant gap in current healthcare systems that needs to be addressed in the future.

At the very end, what remains essential is recognizing the need for transparent and responsible management of conflicts of interest across all stakeholders—patients, scientific societies, journals, pharmaceutical companies, and others. Ideally, all stakeholders should collaborate to establish and adhere to shared principles for effectively addressing conflicts. This would allow an optimal recognition of any underlying influence on their positions beyond legitimate interests. Public transparency in approval and reimbursement discussions would significantly advance this objective.

In conclusion, equitable global access to systemic HCC treatments requires urgent, collaborative efforts from stakeholders to address disparities driven by regulatory barriers, fragmented systems, and high costs, with advocacy playing a key role in raising awareness and influencing policy changes.

## Contributors

Study concept and design: MA, LDF, MSZ, WMC, CM, KL, JB, HLR, RS, BS.

Acquisition of data: MA, LDF, MSZ, WMC, CM, KL, ML.

Analysis and interpretation of data: MA, LDF, MSZ, WMC, CM, KL, ML, SM, ZM, JB, HLR, RS, BS.

Drafting of the manuscript: MA, LDF, MSZ, WMC, CM, KL, ML, SM, ZM, JB, HLR, RS, BS.

Critical revision of the manuscript for important intellectual content: MA, LDF, MSZ, WMC, CM, KL, ML, SM, ZM, MM, JB, HLR, RS, BS.

Statistical analysis: MA.

Study supervision: MA, JB, HLR, RS, BS.

All authors approved the final version of the manuscript.

## Data sharing statement

The data that support the findings of this study are available on request from the corresponding author MA.

## Editor note

The Lancet Group takes a neutral position with respect to territorial claims in published maps and institutional affiliations.

## Declaration of interests

**Manon Allaire** reports speaker fees from Astra Zeneca, Bayer, Abbvie, Gilead, Roche, Sirtex and travel grants from Astra Zeneca, Bayer, and Roche.

**Leonardo da Fonseca** reports speaker fees from AstraZeneca, Bayer, BMS, Knight, MSD, Roche, and Sirtex. Research grant from Bayer.

**Marco Sanduzzi-Zamparelli** reports speaker fees from Bayer, AstraZeneca, and Roche and travel grants from Bayer, BTG, Eisai, and Roche.

**Won-Mook Choi**. None.

**Cecilia Monge**. None.

**Ken Liu**. None.

**Michael Leibfried** is an employee of Genentech, Inc, a Member of the Roche Group.

**Sarah Manes**. None.

**Zorana Maravic** reports that part of the funding for Digestive Cancers Europe comes from Amgen, Astellas, AstraZeneca, Bayer, BI, BMS, Daiichi-Sankyo, Jazz Pharmaceuticals, Merck, Olympus, Pierre Fabre, MSD, Novartis, Roche, Sandoz, Servier, and Takeda.

**Milan Mishkovikj**. None.

**Jordi Bruix** reports consultancy fees from AbbVie, Adaptimmune, ArQule, Astra-Zeneca, Basilea, Bayer, Bio-Alliance, Bristol-Myers Squibb, BTG, Daiichi Sankyo, Eisai, Glaxo-Smith-Kline, Gilead, IKF, Incyte, Ipsen, Kowa, Lilly, Medimmune, Merck, Nerviano, Novartis, Onxeo, Polaris, Quirem, Roche, Sanofi-Aventis, Sirtex, Terumo, Universal DX. Speaker fees from Roche.

**Helen L Reeves** reports consultancy fees from Boston Scientific and Astra Zeneca; speaker fees from Sirtex Medical.

**Riad Salem** reports consulting fees from Boston Scientific, Cook, Sirtex, Terumo, Siemens, Astrazeneca, Genentech, Eisai, Roche, and Becton-Dickinson.

**Bruno Sangro** reports consulting or advisory fees from AstraZeneca, Bayer, Boston Scientific, Bristol Myers Squibb, Eisai, Incyte, IPSEN, Roche, Sirtex Medical, and Terumo; speaker fees from AstraZeneca, Bristol Myers Squibb, Eisai, Incyte, IPSEN, Roche, and Sirtex Medical; research funding (to institution) from Bristol Myers Squibb.
